# Stem Cell Neurodevelopmental Solutions for Restorative Treatments of the Human Trunk and Spine

**DOI:** 10.3389/fncel.2021.667590

**Published:** 2021-04-26

**Authors:** Zachary T. Olmsted, Janet L. Paluh

**Affiliations:** Colleges of Nanoscale Science and Engineering, Nanobioscience Constellation, State University of New York Polytechnic Institute, Albany, NY, United States

**Keywords:** stem cells, differentiation, trunk development, spine development, organoids, gastruloids, assembloids, cell therapy

## Abstract

The ability to reliably repair spinal cord injuries (SCI) will be one of the greatest human achievements realized in regenerative medicine. Until recently, the cellular path to this goal has been challenging. However, as detailed developmental principles are revealed in mouse and human models, their application in the stem cell community brings trunk and spine embryology into efforts to advance human regenerative medicine. New models of posterior embryo development identify neuromesodermal progenitors (NMPs) as a major bifurcation point in generating the spinal cord and somites and is leading to production of cell types with the full range of axial identities critical for repair of trunk and spine disorders. This is coupled with organoid technologies including assembloids, circuitoids, and gastruloids. We describe a paradigm for applying developmental principles towards the goal of cell-based restorative therapies to enable reproducible and effective near-term clinical interventions.

## Introduction

For the entirety of recorded human medical history, injury to the trunk and spinal cord has carried with it the potential for untreatable loss of functional modalities, and severely compromised quality and duration of life (Silva et al., 2014). Human stem cell research has reignited the conviction that meaningful functional recovery is achievable. With recent discoveries and technological improvements, new strategies to advance the application of human stem cells to model or repair the trunk and spine are being rapidly implemented. Previous embryonic models based largely on amphibian developmental biology, resulted in the use of non-ideal differentiation strategies that failed to generate cells with the complete range of axial identities needed along the rostral-caudal neuraxis. One major advance was the identification of a pool of caudal axial progenitor cells *in vivo*, referred to as a neuromesodermal progenitors (NMPs; Tzouanacou et al., 2009), and the *in vitro* implementation to derive and cultivate this cell type from human pluripotent stem cells (hPSCs; Gouti et al., 2014). Suddenly, with NMPs as a starting point, the *in vitro* recapitulation of phenotypes along the entire neuraxis became feasible. This developmental discovery in human embryology is opening the door to an astounding degree of progress for generating and understanding cell types of the trunk and spine. In animal models, these cells applied therapeutically will bring anatomical and physiological matching that is expected to remove or overcome previous barriers to cellular repair including early integration events and host interconnectivity by expressing the appropriate targets. Here we discuss advances in human stem cell biology that is an integral component of human developmental neurotechnologies. We describe the clinical potential of developmental neurotechnologies as they relate to injury and disease processes of the trunk and spine.

NMP models that give rise to the trunk and spine including the central and peripheral nervous systems (CNS, PNS) differ strikingly from the organization of the brain into its interacting systems, such as development of the telencephalon and neocortical regions reviewed elsewhere (Molyneaux et al., 2007; Greig et al., 2013). NMPs are thought to functionally bifurcate into separable neuroectodermal and mesodermal lineages, as well as into neural crest cells (NCCs). *In vitro*, NMPs are being used to generate anatomically matched neural cells for injury repair and applied in advanced culture systems such as multi-lineage gastruloids, circuit organoids, and cortico-motor assembloids. These living human cell platforms allow the interrogation of previously unobtainable stages of human development and the earliest manifestations of disease with unprecedented detail. By highlighting recent advances in our understanding of developmental stages of the spinal cord and the metameric segmentation (Diaz-Cuadros et al., 2020; Matsuda et al., 2020), we reveal how the application of such developmental principles to stem cell research and therapies will benefit clinical outcomes for example in congenital disorders, neuromuscular disorders, and adult spinal cord trauma.

## Developmental Principles: Trunk and Spinal Cord

Below we discuss the discoveries from animal models that formed the basis for insights into human development, from anterior to posterior CNS formation, the discovery of NMPs, ventral and dorsal spinal cord patterning, the design potential of NCCs, and the integral roles of glia.

### Anterior vs. Posterior CNS Development

**Figure 1:**
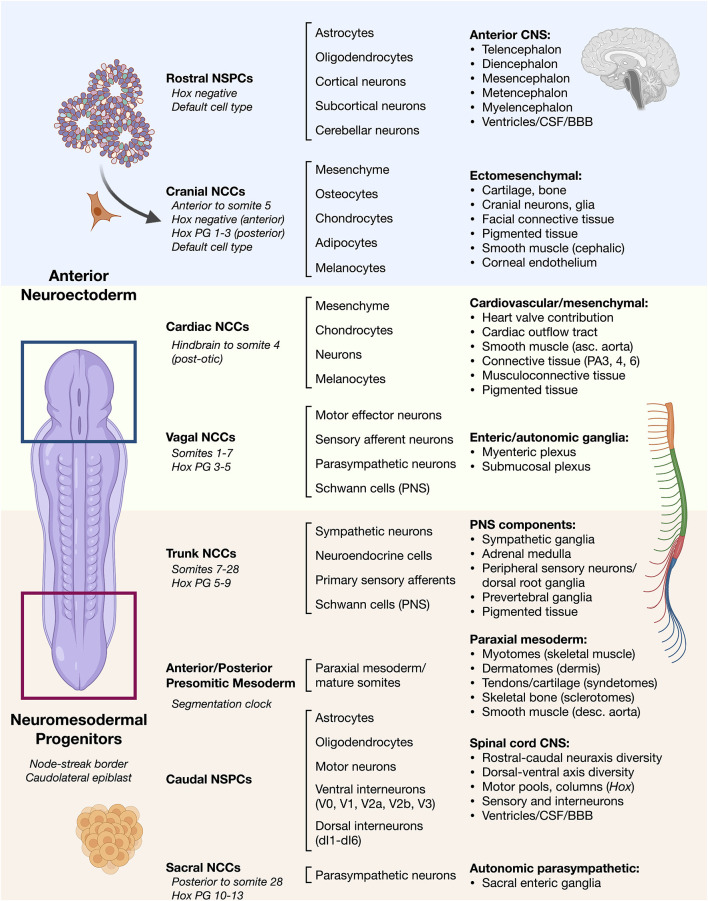
Overview of vertebrate ontogeny of cell types in the head vs. trunk and spine. Anterior/rostral development from neuroectoderm vs. posterior/caudal development from neuromesodermal progenitor cells. Lineage ontogeny from distinct stem and progenitor pools is detailed. General cell sources (left column) and general cell types produced by their differentiation (middle column) along with resulting general tissues (right column) are listed. Differentiation potential varies along the anterior–posterior neuraxis such as in cell types of the neural crest that can otherwise behave similarly.NSPCs, neural stem/progenitor cells; NCCs, neural crest cells; Hox PG, Hox paralogous group; CSF, cerebrospinal fluid; BBB, blood-brain barrier; PA, pharyngeal arch.

In the 1950’s, amphibian studies by Nieuwkoop (Nieuwkoop, 1952; Nieuwkoop and Nigtevecht, 1952) provided the earliest model of nervous system development. In this model, a single pool of neural stem cells (NSCs) arises from early epiblasts in the anterior neural plate and becomes transformed along the anterior-posterior body length by caudalizing, or posteriorizing, signals. This generates the gamut of the rostral-caudal neuraxis from forebrain to spinal cord (Stern, 2001; Andoniadou and Martinez-Barbera, 2013). Foundational vertebrate stem cell differentiation protocols often paralleled this model to produce diverse therapeutic CNS cells for brain and spinal cord applications. However, as a matter of longstanding debate (Handrigan, 2003; Stern, 2006), more recent studies support an extension of the Nieuwkoop model in which the vertebrate brain and spinal cord are instead realized to have independent developmental origins and arise from distinct populations of NSCs (Tzouanacou et al., 2009; Henrique et al., 2015). Accordingly, a foundational study by Metzis et al. (2018) revealed genome-wide chromatin-remodeling events that allow expression of genes for directing “primary axial regionalization.” This revealed an important distinction by regulated expression to specify regionally separate pools of epiblast cells that will be allocated to anterior (forebrain) or posterior (spinal cord) positions in the developing nervous system prior to neural induction. In this revised model for vertebrates, NSCs in the anterior neural plate generate cranial neurons in the brain and descending white matter tracts, whereas induction of distinct posterior axial stem zones begins the generation and patterning of the spinal cord (Figure 1). NMPs are thought to be the common origin of the posterior CNS and its associated musculoskeletal system (Cambray and Wilson, 2002; Wilson et al., 2009) and trigger a cascade of downstream developmental events and pathways. The principle behind therapeutic intervention with human cells derived by stem cell technology is to match cells as accurately as possible to the anatomical site of injury to favor microenvironment integration. For spinal cord injuries (SCI) cell-based therapies, the repair and restoration of function should apply vertebrate posterior developmental processes. Previous use of developmentally-mismatched anterior NSCs for SCI therapy that by default are programmed for the brain creates additional challenges for these cells that must re-align gene expression to adapt to new neurophysiological signaling cues in the spine in a complex cytokine injury microenvironment. NMPs help to remove this barrier.

### NMP Pathways to the Trunk and Spine

**Figure 2:**
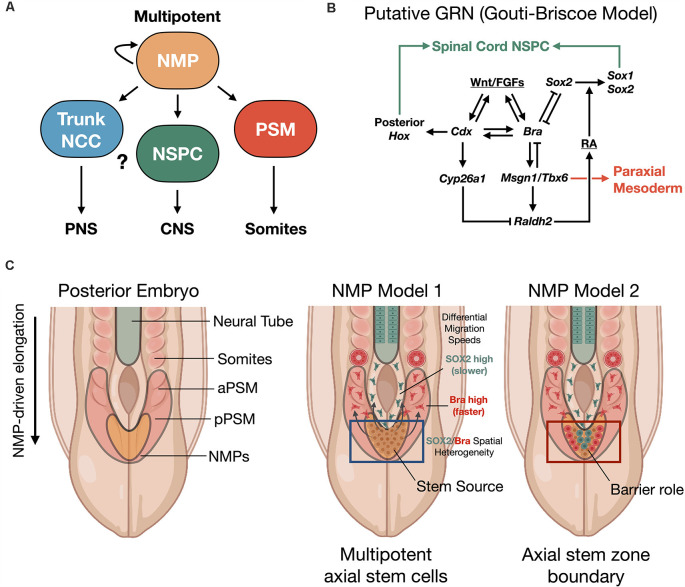
Neuromesodermal progenitor (NMP) models of human trunk and spine development. **(A)** NMPs act upstream as multipotent building blocks to generate the trunk and spine. **(B)** Putative gene regulatory network (GRN) of NMP differentiation (Gouti-Briscoe model). RA (retinoic acid), NSPC (neural stem/progenitor cell). **(C)** Two proposed NMP developmental models. NMP model 1: NMPs in the caudolateral epiblast (CLE)/node streak border (NSB) act as caudal axial stem cells, that give rise to NPCs and the presomitic mesoderm (PSM) that undergoes somitogenesis in waves under a segmentation clock (Henrique et al., 2015). NMP model 2: NMPs form a barrier between distinct neural stem and mesoderm stem zones (Wood et al., 2020). Both models produce the spinal cord and paraxial mesoderm/somites from caudal stem cell pools. SOX2/Bra spatial heterogeneity in NMPs correlates with differential migratory velocity and PSM vs. spinal cord lineage commitment (Romanos et al., 2021). NCC, neural crest cell; aPSM/pPSM, anterior/posterior presomitic mesoderm; PNS, peripheral nervous system; CNS, central nervous system.

Human NMPs constitute a cellular pool with a bipotent fate map for spinal cord and somite development. Their identification reveals that a possible common mechanism in vertebrate embryos investigated from fish to human (Kimelman, 2016). This cell population has not yet been identified in amphibian, which may account for the different findings of Nieuwkoop using the amphibian embryo model system (Nieuwkoop, 1952; Nieuwkoop and Nigtevecht, 1952). NMPs are present within a region called the node streak border (NSB) and from this location contribute to axial elongation of early, developing embryos (Cambray and Wilson, 2002; Tzouanacou et al., 2009; Wilson et al., 2009), sustainably sourcing new neural and paraxial mesodermal tissues (Figure 2). In contrast to Nieuwkoop’s model, in which caudalization of anterior NSCs is sufficient to produce the entire rostral-caudal neuraxis, NMPs appear to contribute predominately to hindbrain and anterior spinal cord, posterior spinal cord, and paraxial mesoderm rather than forebrain and midbrain structures in animal and human models. Thus, NMPs are described as multipotent building blocks of the posterior body (Figures 2A,B) and are reviewed elsewhere (Henrique et al., 2015). In an alternative model that is not necessarily exclusionary, NMPs may also contribute an important role to form a barrier protecting the interface between two developing stem cell zones (Figure 2C; Wood et al., 2020). Molecularly, NMPs are identified by co-expression of mesodermal transcription factor brachyury (T/Bra) and the NSC marker SOX2. Expression of the latter is driven by a unique N1 enhancer element. This enhancer is distinct from the N2 enhancer element that regulates *Sox2* expression in forebrain and in pluripotent stem cells (Uchikawa et al., 2003; Iwafuchi-Doi et al., 2011, 2012). The transition from N2 to N1 enhancer activity correlates with cells that will generate the posterior CNS (Takemoto et al., 2006).

NMPs are transient cell types whose maintenance and differentiation into either presomitic mesoderm (PSM) or into caudal neural progenitors is thought to depend on the spatiotemporal distribution of signaling cues. In particular, inputs of the secretory glycoprotein, Wingless-type integration site protein (Wnt), and fibroblast growth factor (FGF) are interfaced within an intrinsic, complex gene regulatory network (GRN) that we refer to as the Gouti-Briscoe model (Figures 2A,B; Gouti et al., 2017). Briefly, Wnt and FGF signaling are provided by the primitive streak and caudolateral epiblast (CLE) that acts as a caudal neural plate. Here they induce the characteristic SOX2 (N1)/Bra co-expression phenotype. SOX2 levels finely tune this process. Low SOX2 expression enables low-affinity SOX2 genomic binding sites to be occupied by T/Bra and CDX-2 in NMPs (Blassberg et al., 2020). Wnt and FGF also act synergistically to induce *Fgf8* transcription. This further drives a Wnt/FGF positive feedback loop in the posterior embryo (Aulehla and Pourquié, 2008; Wilson et al., 2009). In turn, Wnt signaling activates CDX-2, the upstream master regulator of posterior *Hox* genes that specify body patterning and segmentation (Nordström et al., 2006). The NMP induction is restricted by bone morphogenetic protein (BMP) signaling and by Bra repression of *Sox2* to within the CLE and NSB (Takemoto et al., 2006). Continued exposure to Wnt biases towards mesodermal fate (Garriock et al., 2015), without inhibiting neural cell fates, and drives the further induction of *Hox* genes. The expression of the *Hox* genes is essential to caudal neural development, establishment of neuronal diversity (Sagner and Briscoe, 2019), and ultimately connectivity and circuit organization (Philippidou and Dasen, 2013).

Segmentation into somites from the presomitic mesoderm (PSM) uses the mesodermal transcription factor TBX6 that suppresses *Sox2* *via* the N1 enhancer (Takemoto et al., 2011). Additionally, pairs of somites are rhythmically produced by further control *via* an innate segmentation clock oscillator (Hubaud and Pourquié, 2014). By these mechanisms in zebrafish and mouse vertebrate models, segmented somites form simultaneously and in close relation to the neural tube and as a precursor to functional neuromuscular connectivity. In a recent exciting discovery, Diaz-Cuadros et al. (2020) utilized human stem cell models to demonstrate the existence of this segmentation clock *in vitro* for the first time, which is similarly regulated by Wnt and FGF signaling pathways (Diaz-Cuadros et al., 2020). This segmentation clock was exploited *in vitro* to recapitulate somitogenesis using human PSCs (Matsuda et al., 2020). As somites develop, they express the enzyme retinaldehyde dehydrogenase two that catalyzes the production of retinoic acid (RA; Molotkova et al., 2005). RA gradients are important in defining neuroanatomical regions. By repressing Wnt and FGF-mediated signaling pathways, RA gradients halt the collinear activation of *Hox* genes within the neural tube. This may contribute to neural cell fate commitment with precisely defined positional identity along the rostral-caudal neuraxis during elongation (Diez del Corral et al., 2003; Lippmann et al., 2015). In a new study, it was shown that the NMP population comprising the progenitor zone is spatially heterogenous with differential stochastic expression of SOX2 vs. Bra (Romanos et al., 2021). By *in silico* modeling and *in vivo* validation of the model in quail embryos, it was shown that higher SOX2 expression biases to spinal cord fate while higher Bra expression biases to PSM, where PSM-biased cells have higher migration rates vs. SOX2-biased cells (Figure 2C).

### Dorsal-Ventral Spinal Cord Patterning

The *Hox code* and RA signaling gradients direct rostral-caudal elements of spinal organization (Philippidou and Dasen, 2013). Coincidentally, the regional identity along the dorsal-ventral axis must also be specified and is done so by opposing morphogen signaling gradients that guide neuronal subtype diversification along the dorsal-ventral axis (reviewed in detail: Tao and Zhang, 2016; Sagner and Briscoe, 2019). In the developing embryo, opposing morphogen gradients have long been known to be key concentration-dependent patterning effectors (Turing, 1953; Christian, 2012), and similarly are critical in directing cells of the ventral and dorsal spinal cord to be functionally distinct. The inductive ventralizing Sonic hedgehog (Shh) morphogen is a glycoprotein and growth factor with well-established roles in neural patterning in mouse embryogenesis. Shh is secreted by the notochord at a position that is immediately ventral to the developing neural tube, and later by the floor plate. A signaling gradient is generated that is most highly concentrated at the ventral midline and which diminishes along the ventral-dorsal axis to reliably establish five ventral neural progenitor domains (ventral to dorsal: p3, pMN, p2, p1, p0). These five domains give rise to six functionally distinct neuronal subtypes V3, MN, V2b, V2a, V1, and V0, respectively (Marti et al., 1995; Roelink et al., 1995). Of therapeutic interest for SCI are motor neurons generated from motor neuron progenitors (MNPs) and interneurons that vary in primary neurotransmitter phenotype (broadly, excitatory or inhibitory). Spinal motor neurons (SMNs) are specialized along the rostral-caudal neuraxis into columns. Smaller SMN pools innervate compartments of skeletal muscle or contribute to the autonomic nervous system (Philippidou and Dasen, 2013). Each spinal cord progenitor domain is characterized by a unique combination of transcription factors (Sathyamuarthy et al., 2018; Sagner and Briscoe, 2019), albeit partially overlapping, that provides a molecular signature. Since different transcription factor hetero-complexes activate neuronal subtype-specific genes, how they interact in complex gene regulatory networks and repress alternative transcriptomic developmental pathways is of broad interest. The neural transcription factor code, originally described in nematode (Hobert, 2005–2018), is believed to be critical in vertebrates as well for guiding and specifying neuronal fate (Shirasaki and Pfaff, 2002). Because of the complexity of morphogen gradients and transcription factor codes, the field has not yet linked gene expression molecular profiles with all functionally defined cell types, that includes the full scope of neuronal subtype diversity. The quest for molecular signatures therefore remains a highly active area of research investigation with far-reaching applications therapeutically to provide the most appropriate cell types and supportive networks.

Whereas SMNs reside in the ventral spinal cord, the dorsal spinal cord receives signals from sensory neurons. In a process analogous to ventral spinal cord specification, dorsal spinal cord identity and progenitor domains are also specified by inductive morphogen gradients. Two of these signaling ligand classes, BMP and Wnt, are secreted by the neural tube roof plate (Liem et al., 1997; Lee et al., 2000; Muroyama et al., 2002). They function in opposition to ventral Shh inductive signaling. By short-range interactions between neighboring cells, reliable patterning of region-specific progenitor domains is achieved to ensure high fidelity nervous system connectivity and function. Six dorsal progenitor domains (dorsal to ventral: dp1–dp6) become distinguished through a transcription factor code (Lai et al., 2016; Sagner and Briscoe, 2019). Dorsal progenitor (dp) domains give rise to dorsal interneuron subtypes dI1–6 that use excitatory or inhibitory neurotransmission. This process is thought to be regulated in part by Notch signaling (Mizuguchi et al., 2006). Information flow from the brain *via* descending CNS white matter tracts to the PNS is modulated by synaptic interactions of dorsal interneurons with ventral interneurons and SMNs. The reversed flow of information to the brain from the somatic and autonomic nervous systems through the PNS and spinal cord also relies on interneurons and ascending white matter tracts. Interneurons are core components of sensorimotor neural circuits required for higher-level network integration and behavior. As well, propriospinal neurons that originate peripherally and terminate in the spinal cord and brainstem have been shown to be central for restoring lost locomotor function after SCI (Formento et al., 2018).

### NCCs, PNS, and the Trunk

NCCs constitute a remarkably diverse set of cell lineages that originate at the border of neural ectoderm and non-neural ectoderm in the embryo. The majority of our knowledge about NCCs comes from avian, mouse, and zebrafish models. Unique to vertebrates, NCCs are induced from neuroepithelium at this border and migrate to distant sites throughout the embryo where they differentiate through complex competing pathways and cell state biases into a plethora of mature cell lineages (Etchevers et al., 2019). They are the developmental source of PNS sensory neurons and Schwann cell glia, the enteric nervous system (ENS), the autonomic nervous system, chondrocytes, osteocytes, smooth muscle, melanocytes, and other mesenchymal tissues (Mayor and Theveneau, 2013). Since the first description of single-cell RNA-sequencing (Tang et al., 2009) through to current highly-parallel single-cell RNA-Seq and genome-editing strategies, the many genes and regulatory networks that direct NCC fate specification, epithelial-to-mesenchymal transition (EMT), migration, and differentiation are beginning to be described in context with functional events (Soldatov et al., 2019). Wnt, BMP, and FGF signals mediate the cross-talk between neural and non-neural ectoderm at the neural plate border to specify this region. NCC precursors are distinguishable from neuroectoderm and ectoderm by a suite of neural crest specifier transcription factors that allow molecular lineage tracing. Their position at the edges of the early neural folds changes as the dorsal neural tube closes, and is followed by an EMT that imparts migration capabilities to NCCs to move temporally and peripherally throughout the developing embryo. The ability to recapitulate some of these events in NCC migration and downstream events linking CNS, PNS and enteric nervous systems was realized for the first time, in complex human elongating-multilineage-organized (EMLO) gastruloids (Olmsted and Paluh, 2020).

Migratory NCCs host competing transcriptional programs that are eventually biased to a particular lineage through a series of binary decisions (Soldatov et al., 2019). In mouse, the first of these decisions dictates between sensory and non-sensory fate while subsequent decisions regulate mesenchymal vs. autonomic fate. This study also reported the differential lineage potential of NCCs along the rostral-caudal neuraxis, varying with axial identity. That is, the trunk NCCs have neuronal bias to pattern the PNS while cranial NCCs have mesenchymal bias to produce craniofacial compartments. Strikingly, it was recently shown that cranial NCCs undergo an *in vivo* reprogramming event wherein OCT4 and Nanog are reactivated following ectodermal commitment to then further generate the array of other cell lineages such as mesenchyme (Zalc et al., 2021). This impressive array of known diverse cell types and emerging fate maps emphasize, how important NCC roles are in multiple aspects of neurodevelopment of the PNS. Indeed, non-lethal defects in these processes underlie a spectrum of human deformations, such as in Hirschsprung’s disease, that are deemed neurocristopathies (Etchevers et al., 2019).

### CNS Glia: Oligodendrocytes and Astrocytes

Efforts toward neuro-restoration following injury also consider roles of assisting oligodendrocyte and astrocyte neural cells. The familiar myelin sheath, formed by the interactions of oligodendrocytes with neuronal axons, facilitates signal propagation in neurons, while multiple astrocyte-neuron interactions enable metabolic and neurotransmission homeostatic support and synaptic regulation as part of the described tripartite synapse (Farhy-Tselnicker and Allen, 2018). Studies in animal models reveal that gliogenesis occurs in temporal waves subsequent to early neurogenesis (Rowitch, 2004; Rowitch and Kriegstein, 2010). Oligodendrocyte progenitor cells (OPCs) in mice are produced in two embryonic waves and one postnatal wave. In the first embryonic wave (E12.5 in mice), ventral Shh drives expression of homeobox genes *Nkx-6.1* and *Nkx-6.2* in a gene regulatory network with oligodendrogenic transcription factors *Olig1* and *Olig2* (Vallstedt et al., 2005). Dorsal OPCs are produced in a second, Shh-independent wave (Cai et al., 2005; Vallstedt et al., 2005) that requires BMP and FGF signaling. Oligodendrocytes from dorsal OPCs are retained predominately in the dorsal white matter (Fogarty et al., 2005). In humans, maturation of OPCs to myelinating oligodendrocytes in the CNS continues after birth through the first decades of life, and likely throughout life, and may remyelinate denuded axons (Franklin and Ffrench-Constant, 2008).

Astroglial populations similarly develop in both the brain and spinal cord as heterogenous cell populations (Bayraktar et al., 2015). During the switch from neuronal to glial production in the spinal cord, the transcription factor stem cell leukemia (SCL) expressed in the ventral p2 domain represses OLIG2-mediated oligodendrocyte production to promote astrogenesis. In p1-p3 ventral progenitor domains, the PAX6/NKX-6.1 homeodomain transcription factor code (Hochstim et al., 2008) specifies three subpopulations of GFAP+ astrocytes that vary in molecular profiles. Positional identity is speculated to be coincident with the organizing process underlying functional subtype diversification. Astroglial cells migrate into white matter as VA1–3 populations corresponding to p1–3 progenitor domains along the dorsal-ventral axis. BMP signaling has also been shown to modulate astrocyte development in the spinal cord (Agius et al., 2010). The balance of neurons and glia is therefore necessary in development, and likely also in repair, to support maintenance, maturation, and neural circuitry demands in specific physiological domains for high-fidelity nervous system function. As well, interesting cross-regulation of shared factors like SCL point to additional physiological events that are occurring. Stem cell differentiation models *in vitro* are driving the production of cell types with relevant regional identity needed for optimal functional host interconnectivity after *in vivo* transplantation.

## Development in A Dish: *In Vitro* Stem Cell Differentiation

Early in human development, the formation of the head, trunk and spine requires the interaction of a multitude of cell types. Despite complex multicellular organization, the spatiotemporal processes underlying the growth and patterning of these regions can be separately replicated in part by stem cell differentiation protocols. The convergence of developmental biology, *in vitro* stem cell differentiation, bioengineering technologies, and *in vivo* clinical relevance of trunk and spinal cord diverse cell types together form a strong foundation for developmental neurotechnologies. Multiple previous reviews provide comprehensive descriptions of unique, focused aspects of trunk and spine development (Philippidou and Dasen, 2013; Stifani, 2014; Green et al., 2015; Henrique et al., 2015; Sagner and Briscoe, 2019) and neurotechnologies (Vázquez-Guardado et al., 2020). In the remainder of this review, we describe how developmental principles are being applied to human stem cell research and incorporated with neurotechnologies to advance human therapies.

### Isolating and Decoding Stem Cell-Derived NMPs

**Figure 3:**
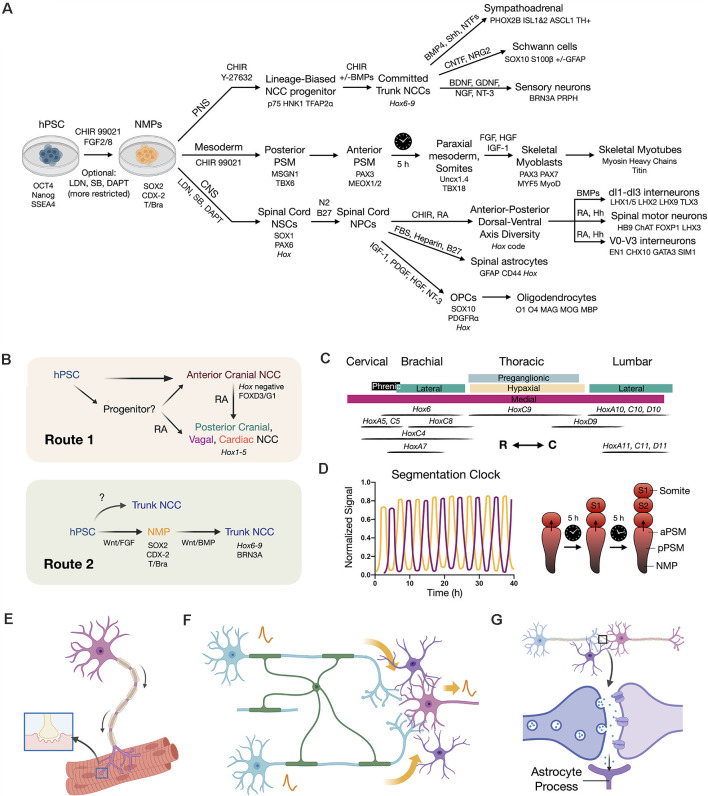
Developmental principles applied to stem cell differentiation. **(A)** Overview flow chart of human pluripotent stem cell (hPSC) differentiation through NMPs. Key differentiation factors and biomarkers are provided. It should be noted that induced protocols with forced transcription factor expression are also used. Variations in differentiation factors for each lineage have been performed in the literature. **(B)** Two routes to caudal neural crest cells (NCCs). Route 1 is to caudalize anterior neural crest progenitors (Hox negative) using retinoic acid (RA) to produce posterior cranial, vagal, and cardiac NCCs (*Hox1–5*). Route 2 is to first generate an NMP intermediate that can in turn produce trunk NCCs with broad axial identity. **(C)** *Hox* code of spinal cord motor columns along the neuraxis (Philippidou and Dasen, 2013). R (rostral), C (caudal) directions. **(D)** Recapitulating the *in vivo* somite segmentation clock *in vitro* (Diaz-Cuadros et al., 2020; Matsuda et al., 2020). **(E)** SMN co-culture with skeletal myotubes to model NMJ function and dysfunction. **(F)** Neural circuit signal propagation efficiency depends on multicellular interactions between neurons (blue/pink), oligodendrocytes (green), and astrocytes (purple). **(G)** Tripartite synapse model wherein astrocytes (purple) assist synapse formation, function, and homeostasis.

Since 2014, *in vitro* studies using mouse and human ESCs have contributed to our understanding of NMP biology. A fortified, extended developmental model for neural induction from separable posterior and anterior stem cell pools is now established (Gouti et al., 2014; Tsakiridis et al., 2014; Turner et al., 2014; Henrique et al., 2015; Lippmann et al., 2015). Gouti et al. (2014) performed one of the most foundational studies on NMPs using human stem cells. The differentiation of mESCs and hESCs to NMPs, done in parallel, as well as findings from Turner et al. (2014) and others (Tsakiridis and Wilson, 2015) demonstrated the bipotential nature of these cells co-expressing SOX2/Bra to produce neuroectodermal and mesodermal cell types *in vitro*. These developmental principles provide a strong foundation for future stem cell therapies to advance repair of SCI with regionally-matched cells (Figure 3A).

In all studies, Wnt agonism by the small molecule CHIR 99021 and soluble recombinant FGF signaling are necessary and sufficient to produce high-yield NMPs. Next generation sequencing technologies are revealing new human temporal information by applying strategies that enable lineage tracing of cell identity during differentiation *in vitro* and comparing this to transitional developmental events (morphological and functional) to capture evolving gene regulatory networks (Figure 2B; Gouti et al., 2014, 2017; Verrier et al., 2018). NMPs are generated by use of CHIR 99021-mediated Wnt agonism plus FGF2 and/or FGF8b in protocols that may include simultaneous BMP, TGF-β, and Notch signaling inhibition for further lineage restriction (Lippmann et al., 2015). This allows the maintenance of cells in an intermediate stage and establishment of stable NSC lines retaining multipotency and spinal cord identity (scNSCs) over multiple passages (Kumamaru et al., 2018). As well, NMPs derived *in vitro* provide a physiologically relevant starting material for production of axial NCCs with trunk identity (Frith et al., 2018).

### Distinct Neuroectoderm Brain and Spinal Cord Lineages

Two pathways to neuroectoderm have recently been defined in mammalian models that define brain and spinal cord lineages of anterior neuroepithelium vs. posterior NMPs. A key advance towards this goal for *in vitro* neural differentiation was the advent of a dual SMAD inhibition protocol utilizing small molecules LDN193189 and SB431542 to inhibit BMP and TGF-β/Activin/Nodal signaling pathways (Chambers et al., 2009, 2012). *In vivo*, the inhibition of pluripotency maintenance pathways involving TGF-β and FGF induces infoldings of the neural plate to form the neural tube. By applying dual SMAD inhibition, NSC generation was both optimized and accelerated due to the repression of stem cell differentiation into non-neural lineages. This produces abundant “neural rosettes” that are approximate 2D representations of the 3D developing neural tube (Conti and Cattaneo, 2010). *In vitro*, this method by default yields NSCs with anterior (forebrain) identity (Pankratz et al., 2007; Rowland et al., 2011). Attempts to subsequently caudalize such NSCs with RA, yields transcriptomic profiles exhibiting activation of 3’ *Hox* genes, that correspond to the hindbrain and cervical spinal cord, but importantly fail to recapitulate the entire rostral-caudal neuraxis (brachial, thoracic, lumbosacral vertebral levels). By applying a method that instead proceeds through an NMP differentiation intermediate *in vitro*, it was shown that caudalization of NSCs achieves more complete *Hox* gene collinearity with expression of 5’ *Hox* genes *HoxD10–12* (Lippmann et al., 2015; Kumamaru et al., 2018; Verrier et al., 2018). This method gained traction in the stem cell field, and was further extended to lumbosacral phenotypes by inclusion of the TGF-β ligand, GDF11 (Lippmann et al., 2015). In the absence of Wnt agonism, the anterior NSC phenotype again predominates (Gouti et al., 2014). In general, longer exposure to graded CHIR 99021 and FGF signaling yields more posterior *Hox* gene induction. RA addition during regionalization *in vitro* is sufficient to arrest this process yielding a population of cells with fixed rostral-caudal identity (Figure 3C; Mazzoni et al., 2013; Lippmann et al., 2015). When exposed to RA signaling, the NMP transcription factor Bra that otherwise biases towards mesoderm is downregulated. This allows entry into neuroectoderm and subsequent neuronal differentiation (Maury et al., 2015). These approaches mirror *in vivo* developmental signaling and outcomes.

One core principle emerging from NMP induction and patterning of the posterior CNS is the requirement for caudalization prior to or in conjunction with differentiation and lineage commitment (Metzis et al., 2018). Throughout elongation of the embryo, RA production by adjacent somites in animals works to establish the *Hox* code for body patterning and segmentation (Diez del Corral et al., 2003; Molotkova et al., 2005; Wilson et al., 2009). For *in vitro* studies to be relevant for *in vivo* use, the requirement for sufficient regionalization prior to neural differentiation must be mimicked. Recently, the *in vivo* action of a segmentation clock associated with waves of somite production was recapitulated *in vitro* using human stem cells (Figure 3D) (Diaz-Cuadros et al., 2020; Matsuda et al., 2020). Together, these multiple important refinements in differentiation protocols enable us now to achieve the production of regionally-specified neuronal and mesodermal subtypes. Spinal cells generated in this manner are expected to be better primed for rapid spinal cord integration in regenerative cell therapies and will be more accurate for *in vitro* disease modeling.

### Realization of MNP and SMN Subtype Diversity for Reproducible Spinal Cord Therapy

The extent of neuronal diversity in the human spinal cord is unknown but expected to be extensive. Among the most highly refined information in this regard comes from stem cell differentiation protocols to SMNs (reviewed in: Davis-Dusenbery et al., 2014; Sances et al., 2016; Trawczynski et al., 2019) and include SMN subtype- and regional-specification (Stifani, 2014; Patani, 2016; Tao and Zhang, 2016). SMN somata residing in the spinal cord ventral horn project axons *via* peripheral nerve conduits to innervate muscle tissue or contribute to the autonomic nervous system (Figure 3E). In contrast, cranial, or upper MNs (UMNs) are those that develop in the neocortex and synapse with SMNs and interneurons *via* descending white matter tracts. In seminal work with mESCs, SMN differentiation was induced through a committed MNP intermediate by withdrawal of leukemia inhibitory factor (LIF) and subsequent stimulation of RA and Shh signaling pathways (Wichterle et al., 2002), a strategy also applied successfully to human ESCs (Li et al., 2005). A more robust protocol is now available that potentiates neuroectodermal programs by combining dual SMAD inhibition to prevent mesodermal and endodermal differentiating contaminants (Chambers et al., 2009, 2012). Currently, dual SMAD inhibition uses LDN 193189 (BMP pathway inhibitor by ALK1, 2, 3, 6 inhibition) and SB 431542 (Activin/BMP/TGF-β pathway inhibitor by ALK4, 5, 7 inhibition). Progress has also been made to further refine SMN subtypes generated by additional perturbation of RA and Shh signaling pathways.

Therapeutically, the ability to ideally match neuron type with physiological need is an exciting and critical advancement. For example, generating SMNs with lateral motor column (LMC) identity that *in vivo* innervate skeletal muscles of the limbs to assist in locomotion (Amoroso et al., 2013). MNP protocols have also extended to human iPSCs (Dimos et al., 2008; Hu et al., 2010), and it is now possible to generate highly pure cultures of MNPs across stem cell models (Qu et al., 2014; Du et al., 2015). An excellent summary of SMN differentiation protocols used across many studies, is detailed in Sances et al. (2016). Most previous transplantation studies with human NSCs have used dual SMAD inhibition alone. This generates NSCs with anterior forebrain identity that must be subsequently caudalized, whereas the recent redirection to first generate spinal cord NSCs through NMPs is a more developmentally matched strategy.

The expectation is that anatomic regional matching will improve functional recovery. This is being done to favor host integration of transplanted cells and to advance *in vitro* disease modeling and drug screening. A clear example is seen with disease progression of amyotrophic lateral sclerosis (ALS), wherein distinct SMN pools are selectively vulnerable to neurodegeneration (e.g., lumbar ventral horn neurons), while others are resistant (e.g., brainstem oculomotor neurons) (Nijssen et al., 2017). This critical finding is recapitulated by developmentally matched differentiation protocols (Allodi et al., 2019). In addition to traditional cell culture and signaling pathway manipulation, alternative protocols with forced viral or mRNA-mediated expression of motor neurogenic transcription factors, and direct conversion from somatic cells, are being explored (Hester et al., 2011; Son et al., 2011). The goal of these trans-differentiation protocols is to achieve high-yield production of functional SMNs in a protracted timeframe. RNA-Seq remains a key strategy to initially compare gene expression profiles of cells generated by these alternative methods before functional testing *in vivo*. In this regard, one substantial barrier in the SCI field is the ability to rapidly access the outcomes of transplanted cells. New technologies such as intravital windows, improved biomarkers for connectivity mapping, and electrical stimulation and readouts are desperately needed to rapidly assess successes or failures and to accelerate the pace to therapeutic intervention.

### Completing Neural Circuits With Ventral and Dorsal Spinal Interneurons

Ventral and dorsal interneurons are components of the precise neuronal circuits needed for spinal cord repair (Francius et al., 2013). These cells have remained understudied, not for lack of importance, but due to difficulties in specifying pure interneuron subtypes. However, by applying NMPs, the ability to begin to model circuit formation event *in vitro* is expanding (Nedelec and Martinez-Arias, 2021). Recent advancements out of the Sakiyama-Elbert lab have enabled new protocols to produce V3 excitatory commissural interneurons using mESCs (Xu and Sakiyama-Elbert, 2015), and V2a excitatory interneurons both from mESCs (Brown et al., 2014) and human PSCs (Butts et al., 2019). V3 interneuron (NKX-2.2 + /SIM1+) differentiation was similar to SMN protocols but modified to decrease RA concentration (10 nM) and lengthen the exposure to the potent Shh agonist, SAG (~18% efficiency NKX-2.2 cells). V2 interneurons originate from the p2 progenitor domain that further subdivides into excitatory (V2a, CHX10+) and inhibitory (V2b, GATA3+) regions as neurons specialize. Notch signaling promotes the inhibitory V2b subtype, but directly inhibits the generation of excitatory V2a neurons. In a recent protocol (Butts et al., 2019), the γ-secretase inhibitor DAPT was added early in neural induction to inhibit Notch, and along with dual SMAD inhibition and optimized RA/Shh concentrations was sufficient to bias towards V2a excitatory interneuron fate. These advancements begin to open up studies and therapeutic applications of interneurons.

As with ventral interneurons, detailed methodologies for dorsal interneurons have lagged behind SMN advances, but are beginning to emerge. These protocols similarly co-opt developmental principles governing dorsal spinal cord patterning into distinct progenitor domains dp1-dp6, and specialization into laminae containing the corresponding interneurons dI1-dI6. The first protocol for generating dorsal spinal interneurons was published in 2018 (Gupta et al., 2018). Gupta et al. (2018) leveraged BMP4 and RA signaling to dorsalize and caudalize neural progenitors, respectively. This method generates dorsal interneurons of three classes that are dI1, dI2 (proprioceptive) and dI3 (mechanosensory). A critical feature of this method was the requirement for optimal timing of BMP4 exposure in a temporally-restricted window to guide neural progenitors towards dI1 and dI3 fates. Unexpectedly, dI2 neurons were only observed in RA control cultures and were suppressed by exposure to BMP4. This protocol was applied to both hESC and hiPSC models demonstrating a similar developmental program and timeline for dorsal sensory interneurons. Dorsal interneuron production has also now been extended to 3D organoid culture platforms (Ogura et al., 2018; Duval et al., 2019). Neural organoid exposure to BMP4 was sufficient to recapitulate characteristic arrangements of dorsal neural tube cells which are capable of differentiating into patterned subsets of dorsal interneurons using both mouse and human stem cell models (Duval et al., 2019). As these protocols are refined, cell replacement strategies in SCI, where acute injury can impact both motor and sensory systems, will benefit. Propriospinal neurons remain of particular interest since they convey and integrate positional feedback information, and are essential for restoring behavioral locomotor function in animals and humans (Formento et al., 2018).

### NCCs Guide Axial Identity and CNS Spinal Integration With the PNS

Multipotent NCCs share transcriptomic similarities to PSCs and have an impressive capacity to produce a diverse array of adult cell types such as peripheral sensory and autonomic neurons, Schwann cells, enteric ganglia, bone and cartilage, pigmenting melanocytes, sympathoadrenal cells, smooth muscle, and other cells of the mesenchyme in the trunk and craniofacial anatomic compartments (Srinivasan and Toh, 2019). Given the multipotent nature of NCCs, differentiation protocols for generating terminal cell types through NCC intermediates are numerous. While early protocols required stromal feeder layers (Pomp et al., 2005; Lee et al., 2007) or employed suspension neurospheres (Pomp et al., 2008), these strategies helped to enable differentiation to PNS sensory and sympathetic neurons (Pomp et al., 2005; Brokhman et al., 2008). It is now more common to use fully defined human differentiation protocols to generate NCCs and downstream cells (Menendez et al., 2011; Mica et al., 2013; Fattahi et al., 2016). Exposure of NCCs to BMP2/4, Shh, or FGF can drive the production of roof plate, floor plate, and neuroepithelial tissue, respectively (Denham et al., 2015). The caudalization by RA is employed when patterning more posterior trunk NCCs as opposed to those with anterior craniofacial identity (Huang et al., 2016), reflecting lineage biases that differ between trunk and craniofacial compartments (Soldatov et al., 2019). Direct transcription factor-based reprograming methods to generate induced NCCs, using *Sox10* or *FoxD3* forced expression have also been developed (Kim et al., 2014). Continued refinement of protocols for NCCs that are regionally patterned along the rostral-caudal neuraxis will be critical to complex regenerative repair of spinal damage. Damage that extends beyond CNS tissue will also require new bone, cartilage, tendon, peripheral nerves, autonomic neurons, and myelinating Schwann cells (Srinivasan and Toh, 2019).

The axial positions of NCCs are reflected in the *Hox* gene code and correspond to differential capacities in lineage specification (Frith et al., 2018). While Wnt signaling dictates the cranial vs. trunk decision, FGF signaling modulates axial identity (Hackland et al., 2019). *In vitro* NMPs have been used to produce sympathoadrenal progenitors and sympathetic neurons (Kirino et al., 2018; Saito-Diaz et al., 2019), as well as enteric neural progenitors and neurons (Frith et al., 2020). Knowledge of how to differentiate NCCs with a given axial fingerprint is advancing. NCCs by default, without signaling interventions, produce primarily anterior cranial NCCs that are *Hox* negative. Similar to the situation in the CNS, two routes exist to produce NCCs with more caudal identity (Figure 3B). The first uses RA to caudalize anterior cells to posterior cranial (*Hox1–3)*, vagal and cardiac (*Hox3–5*), but not trunk, NCCs. The second route is through caudal NMPs to produce NCCs with trunk identity (*Hox6–9*). At present, this approach seems to be the sole method of generating trunk phenotypes and is paralleled in CNS protocols. The recognition of the central role of NMPs even in regards to NCCs has therefore been a major turning point in understanding and treating the trunk and spine.

Sensory neurons in the PNS are broadly specialized for sensing noxious, thermal, mechanical, or proprioceptive stimuli and relay this information to the CNS directly and through connections with dorsal spinal interneurons (Lai et al., 2016). Sensory neurons are characterized based on electrophysiological phenotypes, axon diameters, and surface receptor expression, and are also categorized by sensory modality. Early strategies to produce nociceptive sensory neurons again used neuroectodermal induction through dual SMAD inhibition followed by nociceptor induction by inhibitory small molecules CHIR 99021, SU 5402, and DAPT (Chambers et al., 2012; Clark et al., 2017). More recently, strategies aimed at achieving a closer adherence to the developmental origin of the PNS apply NCC intermediates from hPSCs to generate diverse sensory neuronal subtypes found within the dorsal root ganglion (Denham et al., 2015; Alshawaf et al., 2018). Alshawaf et al. (2018) optimized the temporal addition of inhibitors and soluble ligands to produce sensory neurons that respond to noxious, thermal, and mechanical stimuli.

### A Critical Role for Support Cells in Meeting the Therapeutic Threshold

SCI cellular repair may require a balance between retaining existing neurons and the need to replace lost neurons. Oligodendrocytes play critical support and structural roles for CNS neurons, enabling saltatory conduction, trophic support and axonal stability (Figures 3F,G; reviewed in Li and Leung, 2015). They are the myelinating cells for CNS axons within the subpial white matter, whereas neurons with axons directed distally to muscles utilize specialized Schwann cells for myelination. Studies in animal models following SCI demonstrate acute (Grossman et al., 2001) and chronic (Totoiu and Keirstead, 2005; Lytle and Wrathall, 2007) loss of CNS myelination. Demyelination following SCI trauma and the challenge for host oligodendrocyte progenitor cells (OPCs) to activate and fulfill the massive post-traumatic remyelination needs has led to a desire for therapeutic strategies that generate and co-transplant OPCs with other spinal cell types to protect against chronic die-back of axons and facilitate regeneration (Li and Leung, 2015). In 2005, the high-efficiency production of OPCs from hESCs *in vitro* and ability to promote remyelination with OPC transplants with improved locomotion in rodent models of SCI demonstrated one strategy to further optimize cell-based regenerative therapies for SCI (Keirstead et al., 2005; Nistor et al., 2005). This foundational work formed the basis for the first human clinical hESC trial for SCI by Geron (Geron Corporation, 2009; Scott and Magnus, 2014). Although the Geron study was discontinued, in part due to costs, in subsequent years advances in the *in vitro* production of OPCs and oligodendrocytes have continued (reviewed in Goldman and Kuypers, 2015). This is now being coupled with significant advances in generating homotypic spinal cell types relevant to the site of injury that together with OPC studies have invoked a new era of near term optimism for the clinical use and success of stem cell based neural therapies.

In hESCs, research has shown that the vertebrate developmental signaling pathways to sequentially activate oligodendrogenic transcription factors, bHLH-type OLIG2/NKX-2.2/SOX10 *via* Shh can be replicated, and along with other factors drive OPC maturation and myelinating potential. Initially, FGF2 is used to stimulate pre-OPC induction and expansion, but then later must be removed since it acts to inhibit further differentiation to oligodendrocytes by repressing the OLIG2 and NKX-2.2 expression induced by Shh (Hu et al., 2009). Oligodendrocyte protocols are now substantially refined by many groups (Goldman and Kuypers, 2015) and incorporate common principles including temporal exposure to FGF/RA conditions prior to stimulating the Shh pathway. In addition to FGF2, platelet derived growth factor (PDGF) exposure also helps to drive expansion of OPCs *in vitro* while insulin-like growth factor 1 (IGF-1) and thyroid hormone are added to promote maturation to myelinating oligodendrocytes. Three key articles demonstrate that successful sequential developmental events in fully defined systems to produce OPCs and oligodendrocytes *in vitro* that are capable of functional activity in rodent myelination models (Wang et al., 2013; Douvaras et al., 2014; Piao et al., 2015). These protocols are detailed in the literature (Douvaras and Fossati, 2015). Both hESC- and hiPSC-derived OPC populations have been generated at ~70–80% efficiency as validated by a panel of biomarkers. Protocols have also been developed that generate induction by small molecules to mimic normal development. These strategies are all currently time-inefficient however, requiring up to 150 days to produce functioning oligodendrocytes. This may reflect the natural time course of oligodendrocyte development in human gestation in oligodendrogenic waves. An alternate approach by Yang et al. (2013) instead applied direct lineage conversion of fibroblasts to oligodendrocytes by forced expression of SOX10, OLIG2, and ZFP536 in a protracted time course. To study oligodendrocyte maturation along with interactions with other neural cell types *in vitro* over extended time periods, a defined 3D spheroid culture and differentiation protocol was recently established (Marton et al., 2019). These oligodendrocytes shared electrical properties with mature *in vivo* counterparts including the firing of glial action potentials. A single oligodendrocyte can myelinate up to seven neurons. The use of *in vitro* models along with continued animal studies will better define the number of OPCs needed to transplant in context with the SCI features for therapeutic recovery.

As in the brain, astrocytes are important for neural homeostasis in the spinal cord. Brain astrocytes were first produced from hESCs in 2011 (Krencik and Zhang, 2011), requiring 6 months to generate pure cultures. These lengthy initial protocols yielded cells with immature reactive phenotypes, generated from anterior NSCs. A few years later Roybon et al. (2013) detailed a method for inducing astrocytes with spinal cord identity from mESCs, hESCs and hiPSCs with the ability to transition between immature reactive and mature quiescent phenotypes. Following developmental principles, RA-mediated caudalization and ventralization in the context of dual SMAD inhibition was exploited with the addition of ascorbic acid. Notably, FGF1 or FGF2 was sufficient to induce a mature, quiescent phenotype while pro-inflammatory cytokines TNF-α and IL-1β induced reactivity, marking a key advancement in the study of dynamic and extrinsically-influenced spinal astroglial phenotypes *in vitro*.

### Gene Editing and Whole Transcriptome Sequencing: From Bulk to Single Cells

Since the CRISPR/Cas9 system was adapted to mammalian cell types in 2013 (Cong et al., 2013), there has been an explosion in the ability to edit genomes to developmental and therapeutic ends including for the CNS (Zavvarian et al., 2020). The application of CRISPR and other genetic and optogenetic approaches for SCI treatment was recently expertly reviewed (Paschon et al., 2020). Beyond error-correction for diseases, the power of CRISPR is in setting up biosensors to address the critical need in the SCI field to identify the legitimate integration between host tissue and transplanted therapeutic cells, as well as function such as by calcium-encoded readouts, neuronal activity such as cFos, and synaptogenesis. Towards this end, CRISPR along with optogenetics and other tools will bring reproducibility and analysis to the field. In addition to gene editing, further linking cellular phenotypes and functions to gene expression has long been a driving force in biology, and in recent decades the development of next generation sequencing tools has ushered in an explosion of information as well as of methodologies and analysis platforms to manage whole-transcriptome data. Initially, differentiating stem cell cultures were characterized using bulk RNA-Seq in which a library of RNA is prepared from an ensemble of heterogenous cells. The resulting picture is a one of a population average of global gene expression levels. Although extremely valuable, this technique does not benefit from single-cell resolution and therefore fails to detect stochastic variation in gene expression even between two cells that are otherwise considered identical. Developmental biology and stem cell fields have been revolutionized by the advent of single-cell RNA-Seq techniques that enable the elucidation of cell identity, transition states, and heterogeneity over time during development, reprogramming and differentiation (Kulkarni et al., 2019), otherwise known as fate mapping. Highly-parallel single-cell RNA-Seq permits the detection of rare cell populations in a differentiating culture and also identifies key transitional phenotypes that are critical to cell fate determination and ultimately to terminal differentiation potential. These methods help to delineate the identities and proportions of diverse cell types in a given heterogenous population by cluster analysis, and have been particularly applicable to organoid studies in which complex cytoarchitectures self-organize as models of or precursors to functional tissues. Further analysis by ATAC-Seq (assay for transposase-accessible chromatin using sequencing) or ChIP-Seq (chromatin immunoprecipitation sequencing) informs on chromatin states such as accessibility to transcription factors and identifies binding sites for proteins that complex with nucleic acids, respectively (Yan et al., 2020). By combining single-cell RNA-Seq with *in situ* cell-omics of intact tissues (e.g., MERFISH, multiplexed error-robust fluorescence *in situ* hybridization), spatial resolution of single cell transcriptome profiles with intact cultures and tissues can be achieved while retaining structural information (Mayr et al., 2019). In one key human study (Ho et al., 2016), single-cell RNA-Seq was used to understand and compare human motor neuron differentiation from iPSCs to fetal tissue counterparts during development. This elegant work resolved gene regulatory networks and pathways at play in differentiating human motor neurons as they mature and age. Ho et al. (2016) also established a framework by which to assess motor neuron functional maturity that contributes to the refinement of differentiation protocols and *in vitro* culture practices. Such studies are expected elucidate key developmental mechanisms that can be mirrored or perturbed in stem cell differentiation to achieve functional maturity. Advances in gene editing technologies and single cell sequencing with retained structural information are seemingly advancing by the day.

## From Stem Cells to Functional Models

**Figure 4:**
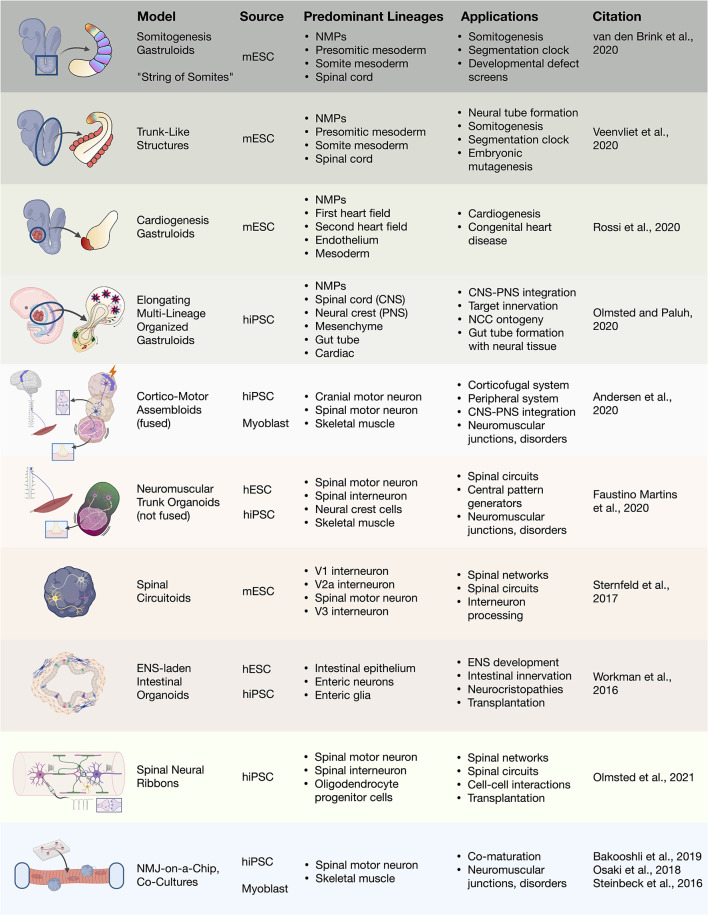
3D heterogenous cell culture developmental models and biotechnologies. Overview of stem cell integration with biotechnologies to produce developmental neurotechnologies. *In vitro* 3D heterogenous cell culture systems include gastruloids, organoids, and assembloids that can be merged with biomaterials, microfluidics, and devices. mESC, mouse embryonic stem cell; hiPSC, human induced pluripotent stem cell; hESC, human embryonic stem cell; NMJ, neuromuscular junction.

The extraordinary capability of pluripotent and multipotent stem cells to self-organize as 3D aggregates that recapitulate mammalian tissues, sub-organ microenvironments, and developmental stages is astonishing and is advancing strategies for medical treatments. The differentiation and maturation of these stem cell aggregates that respond to internal and external cues, such as described in this review, yields so-called “organoids” that can be coaxed into increasingly more complex and functional models. Early developmental events that underlying normal physiology and disease processes are advancing rapidly (Figure 4; expertly reviewed in Amin and Paşca, 2018). For the maturation of multiple neural and non-neural cell types, long-term survival of organoids in suspension (>2 years) has been achieved. Most exciting is the ability to interrogate complex cell-cell interactions in a microenvironment approximating embryological and postnatal events. A fundamental challenge to generating larger organoids beyond millimeter scale, and eventually organs or multi-organ systems, is a distinct lack of vascularization and the homeostatic and signaling benefits that vascular supply can provide. Beyond methods such as transplantation of organoids into mouse models to achieve *in vivo* vascularization and maturation, multiple groups are addressing this *in vitro* by generation of endothelial cells in organoids. Cakir et al. (2019) expressed a gene for the transcription factor master regulator, ETS variant 2 (ETV2), in human cortical organoids to achieve vascular-like networks (Cakir et al., 2019; Grebenyuk and Ranga, 2019). However, whether this is sufficient to recapitulate the dynamic spatiotemporal developmental interplay along with multiple other differentiation events in tissue specific context is unclear (Grebenyuk and Ranga, 2019), and organoids still expand to large millimeter-scale dimensions without a vascular contribution.

### Gastruloids, Circuitoids, and Assembloids

During embryonic development, the earliest stages of organogenesis that may be helpful for understanding morphogenetic patterning and regeneration in the human trunk and spine are morally challenging to study. In 2014, remarkable progress was made using mESCs to recapitulate 3D neural tube patterning (Meinhardt et al., 2014), symmetry breaking, and axial organization in aggregates (van den Brink et al., 2014; Figure 4). In the later study, the use of small cell number aggregates (300–400 cells) yielded symmetry-breaking stochastic events and subsequent non-linear polarized growth (van den Brink et al., 2014; Baillie-Johnson et al., 2015), critical embryonic development events. Amazingly, self-organized “gastruloids” form multiple germ layers and in the absence of extra-embryonic tissues (Turner et al., 2017; Beccari et al., 2018; Rossi et al., 2021). Gastruloids have enabled our ability to study embryonic mechanisms for anterior–posterior polarization, axial elongation, and compartmentalized signaling without the use of embryos. Attention to developmental signaling events created improved protocols, and along with Matrigel embedding, coaxed formation of trunk-like structures containing a neural tube with segmented mesodermal somites (Veenvliet et al., 2020) as well as extended somitogenesis in gastruloids (van den Brink et al., 2020). The ability to use gastruloids to integrate central spinal cord and peripheral NCC-derived neurons was recently demonstrated in elongating multilineage organized (EMLO) gastruloids (Olmsted and Paluh, 2020). The EMLO gastruloids are formed from SOX2/FOXA2 mesendoderm and SOX2/Bra NMP starting cells and encompass features of both central and peripheral nervous systems including the enteric nervous system. This novel platform enables the multi-lineage study of nervous system development, NCC ontogeny, and neuromodulators. In the trunk and spinal fields, gastruloids now represent a powerful embryo-like system in which to study fundamental human multi-lineage developmental processes with tremendously broad neurotechnology implications.

Heterogenous 3D tissue models also enable the interrogation of neural circuitry. Rhythmically-active spinal circuits have been generated within 3D aggregates. Sternfeld et al. (2017) incorporated mESC-derived spinal motor and interneurons into aggregates, deemed “circuitoids,” for the dissection of neural circuit formation and function. Human stem cell based models that are 3D suspension cultures provides a method to drive the maturation of multiple neuronal types, particularly of motor neurons (Rigamonti et al., 2016). Dorsal or ventral morphogen patterning of 3D aggregates was shown to be sufficient to spontaneously form distinct human progenitor spinal domains prior to interneuron differentiation (Ogura et al., 2018; Duval et al., 2019). Andersen et al. (2020) recently recapitulated the functional human cortico-motor tract by the fusion of three regionally-patterned spheroids into sophisticated “assembloids.” As well, the assembloid approach was used in enteric nervous system development by seeding human intestinal organoids with hPSC-derived NCCs to produce a rhythmically-active gut-neural assembloid (Workman et al., 2017). By use of *in vivo* grafting further maturation of the gut-neural assembloid was achieved, including generation of neuroglial structures resembling myenteric and submucosal plexuses as well as functional interstitial cells of Cajal, and electromechanical coupling to regulate contraction wave propagation. This ability in multiple mouse and human models to address neural circuitry, CNS and PNS co-development and interplay, and multi-lineage organogenesis will be extremely beneficial to help direct therapeutic cell types and regenerate functional networks. These studies also raise interest in approaches to trunk and spine therapy in regards to optimal transplanted cellular content. Different considerations include NSCs, directed spine neural progenitors, or more complex anatomically matched cells and circuitry to accelerate repair and reproducible recovery. Along with functional recovery, the ability comes to regulate spinal mechanisms of pain, recently achieved by transplantation of inhibitory interneurons (Manion et al., 2020).

### Neuromuscular Models in Organoids and Bioengineered Platforms

Functionally-active neuromuscular trunk organoids from hPSC-derived NMPs were recently achieved (Faustino Martins et al., 2020), that build on previous key studies (Gouti et al., 2014; Warmflash et al., 2014). These informative neuromuscular trunk organoids contain an astonishing myriad of differentiating cells types and their progenitors, including SMNs, spinal interneurons, skeletal muscle, myelinating glia, astrocytes, and neural crest-derived tissues such as sclerotome/cartilage cells and Schwann cells. The neuroectodermal and mesodermal tissues self-organize into distinct functionally connected compartments. The trunk organoids exhibit posterior *Hox* profiles (*HoxC9–10*) by single-cell RNA-Seq, and mature structural features like myelinated neuronal fibers and functional neuromuscular junctions (NMJs). Central pattern generator-like circuits formed spontaneously, with skeletal contractions driven by electrically active neurons. These organoids are being used to model pathophysiological aspects of myasthenia gravis, a neuromuscular disease that later manifests in muscle weakness in walking and facial movements. Human disease lines generated from patient iPSCs will be particularly useful in modeling CNS or neuromuscular diseases with organoids, gastruloids, assembloids and circuitoids. This approach has already revealed novel mechanisms in disease pathology for spinal muscular atrophy and familial amyotrophic lateral sclerosis (ALS; Sances et al., 2016; Adami and Bottai, 2019).

Beginning with simpler systems and integrating human stem cell organoid technologies (Figure 4), spinal and neuromuscular physiology is benefiting from bioengineering. This includes multilineage tissue engineering for multiple sclerosis (Maffioletti et al., 2018) and neuron optogenetics for ALS and the neuromuscular junction (Osaki et al., 2018; Bakooshli et al., 2019). Engineered devices enable the cost-effective, reproducible, customizable, and scalable high-throughput analysis of reductive cell–cell interactions in defined systems. These can be further integrated with optogenetic or electrical controls (Steinbeck et al., 2016). Kawada et al. (2017) used patterned microchannel devices to generate motor nerve organoids that project a unidirectional fascicle of axons. Tissue engineered *in vitro* platforms are also useful in modeling complex injury environments, such as with SCI, wherein highly parallel experiments can be conducted to refine the conditions translated to cost-intensive animal studies. These platforms can be fully reconstituted from *in vitro* components, or can incorporate spinal cord explant slices as *ex vivo* models of injury (Weightman et al., 2014). In a recent study, Giandomenico et al. (2019) integrated human organoids and mouse *ex vivo* spinal explants. The cerebral cortical organoids formed at the air-liquid interface projected extracortical pyramidal-like tracts that innervated spinal explants to induce muscle contractions. In effect, this study demonstrated recreation of the entire cortical-spinal-muscular circuit containing human upper motors neurons and mouse spinal motor and interneurons. This effort was also successfully demonstrated in a fully human stem cell system (Andersen et al., 2020). Such studies demonstrate the incredible power and pace at which *in vitro* systems that can be combined with biomaterials to impart higher organization in customizable geometries for *in vivo* transplantation and restoration of function after injury.

## Human Stem Cell Technologies and Clinical Challenges

### A Brief History of CNS Grafts for SCI Therapy

The vertebrate CNS has historically been regarded as a non-regenerating organ system, and spinal cord injury “an ailment not to be treated.” The history of SCI treatments has been reviewed elsewhere (Silver, 2005; Donovan, 2007). Seminal early research to graft intact fetal or adult tissue into the injured CNS provided foundational evidence that restoration of damaged circuitry by exogenous therapeutic cells is feasible (Das, 1990; Ramon y Cajal et al., 1991). Prior to the late 21st century, these models were applied to advance basic neuroscience studies. This included the investigation of intrinsic and extrinsic barriers to spontaneous regeneration of CNS neurons, high-fidelity axon path finding and target innervation, the wiring and re-wiring of the CNS, and interactions with ECM and other signaling ligands that influence these processes. The concept of “bridges” was also explored, in which a nerve graft was transplanted in a rat with intact spinal cord to attempt to link medullary and thoracic regions (David and Aguayo, 1981). This model system allowed transplant-host bridge junctions to be tracked with relative ease. Host spinal axon extension into the peripheral nerve grafts within the CNS was observed, but the study also revealed possible inhibitory effects from the host injury environment. Early transplant work also assessed the viability and survival of fetal grafts in the injured CNS in rodent models. Beginning in the 1980s, Bregman and Reier (1986) demonstrated that fetal spinal cord grafted into the mid-thoracic region of <72 h postnatal rats can retain some degree of viability, differentiate and integrate to establish graft-host synaptic connectivity (Reier et al., 1986; Bregman et al., 1993). In 2001, fetal grafts were shown to be safe and feasible in humans (Wirth et al., 2001) in a Phase 1 study. However, ethical concerns limit broad use of such therapies. The emergence of the mouse stem cell field in 1981 (reviewed in Evans, 2011) and later in human stem cells in 1998 (Thomson et al., 1998) has refocused efforts on stem cell based therapies.

Initial successes influenced a transition to cellular-based therapies in SCI (reviewed in Assinck et al., 2017) to promote neuroregeneration, neuro-restoration and functional improvement. Mesenchymal stem cells (MSCs) have a long history in transplantation (reviewed in Cofano et al., 2019), as well as other primary candidate cell types such as olfactory ensheathing cells and Schwann cells. More recently OPCs, NSPCs, lineage-restricted progenitors, and in some instances, postmitotic neurons have seen increased use (Assinck et al., 2017; Lee et al., 2020). In 2006, the advent of reprogramming to induce pluripotency (Takahashi and Yamanaka, 2006) and improved differentiation protocols accelerated human neural stem cell endeavors, as it became possible to establish patient-specific, renewable cell resources for grafting that have tailored neural identities and reduced immunogenicity concerns (Trawczynski et al., 2019). In order to make clinically relevant cellular therapies more readily accessible and to more accurately model disease, stable population-diverse iPSC lines were derived from donors of numerous self-reported ethnicities (Chang et al., 2015; Tomov et al., 2016). Genome-wide association (GWAS) studies are assisting in elucidating the underlying genetics (Gurdasani et al., 2019). An expansive knowledge base of stem cell therapies with a variety of cell types, injuries, and animal models for SCI has been generated, particularly over the past 20 years. Never before has the SCI field been better poised to develop meaningful and reproducible therapeutic regimens with a revitalized expectation that partial or full functional recovery and restoration of lost modalities is achievable. Meanwhile, specific methodological, technical, and pathophysiological barriers continue to be addressed to refine new approaches.

### Clinical Challenges for Stem Cell-Derived Grafts

The clinical relevance of cell therapies to SCI has remained limited due to a number of pathophysiological barriers inherent to SCI (Kraus, 1996) as well as technical barriers requiring improved methodologies for CNS cell delivery and retention at the injury site. At present, the restoration of spinal cytoarchitectures to a pre-injury state, such as restoring neural circuits, has not been achieved except by a limited extent in pre-clinical animal studies. Neuronal relays that connect rostral, injured axons to caudal targets by use of novel intermediary graft architectures have also been described in numerous studies. However, efficient and accurate transmission of information within a new, non-specific network is challenging, including reversal of signals from excitatory to inhibitory, misdirected, or prematurely dissipated signals. A recent variation on this strategy is generation *de novo* from human stem cells a transplantable neuronal network provided in degradable alginate neural ribbons (Olmsted et al., under review). To obtain physical continuity of any graft, the type of parameters to be optimized has remained fairly constant, but the means with which they are characterized has been extended with additional complexity. Therapeutic neural cell delivery requires region-specified neural cell type(s) and ratios, use of highly-characterized cell sources that include transcriptomics, and appropriate cell dose as well as delivery mechanisms for cell positioning, alignment, and retention at the injury site. Directed differentiation or maturation must be tracked to distinguish transplanted cells from host cells and the degree of integration with host tissue. Earlier studies established precedent that injection of unprotected cells in suspension into the injury site resulted in significant loss of those cells due to the immune cytotoxicity and lost support cells of the microenvironment, and that age of the injury as acute, subacute or chronic was also relevant.

Astrocyte hypertrophy-hyperplasia further fuels the establishment of the glial “scar” that is inhibitory to axon regeneration, although pro-regenerative features of the response mechanism are being revealed (summarized in Bradbury and Burnside, 2019; Yang et al., 2020). The proliferative ability of grafts is a double-edged sword, desired to ensure that sufficient cells are available to heal the injury site, accounting for some cell loss, while avoiding tumorigenicity, such as in neuromas, can occur with NSCs. Tumorigenicity appears reduced in non-human primates vs. rodents (Rosenzweig et al., 2017), and is affected by the maturation state of the graft. With the capability now of human stem cell technologies to provide injury-matched SMNs and support cells, the focus can move to refining biomaterials, microenvironment regulators, and biosensors to overcome pathophysiological barriers. This is expected to provide greater reproducibility and predictive responses that is needed to propel the SCI field into a new realm of opportunities for therapeutic advancement.

The developmental pathways to precisely generate *in vitro* specific cells and tissues of the spine as defined physiologically, functionally and by transcriptomics are in hand and expected to work their way into the clinic. More recent extensions of these technologies include assembloids and gastruloids, as well as possible small molecule interventions to avoid transplantation altogether. The fundamental *in vivo* pathophysiological mechanisms underlying SCI are also being targeted therapeutically to promote regeneration. It appears now as a question not of *if*, but *when*, full restoration of lost functional modalities after injury will be achievable. The merger of therapeutic cells with biomaterial scaffolds, neurotrophins and growth factors, and injury site enhancers such as the bacterial enzyme chondroitinase ABC are summarized (Katoh et al., 2019).

The *in vivo* direct lineage conversion and activation of endogenous NSCs is an alternative to the replacement of injured cells in the CNS. One approach applies recombinant cytokines to promote host tissue regeneration by stimulating endogenous stem cells pharmacologically (Lowry and Temple, 2007; Hachem et al., 2020). Alternatively the *in vivo* direct lineage conversion of somatic cells to neurons by forced expression of transcription factors could overcome endogenous NSC limitations. *In situ* reprogramming to neurons and oligodendroglia from astrocytes in the injured CNS has been demonstrated and reviewed in the literature (Li and Chen, 2016). This approach benefits from minimally-invasive delivery and bypasses immune-rejection concerns, though will also require cGMP-grade production of viral expression vectors. Such approaches rely on what is typically seen *in vitro* with organoid research, which is the inherent ability of cells to differentiate and organize effectively given the needed cues to bypass developmental pathways but achieve sufficient terminal cell identity.

### Large Animal Studies and Human Clinical Trials for SCI

Successful outcomes in the treatment of SCI in rodents and other small animal models that include partial restoration in transected spinal cords has led to the progression to large animal studies (reviewed in Gabel et al., 2017) and early clinical interventions in humans. An exciting combinatorial approach by Lai et al. (2019) applies an acute model of SCI that is a complete thoracic transection using a preconstructed neural network established in collagen scaffolds as a bridge. The neural networks are formed *in vitro* from cells obtained from newborn beagles that are hippocampus-derived NSCs and differentiated into neurons as well as with isolated Schwann cells. This approach encompasses neurotechnologies and scaffolds in an advanced approach that realized repair outcomes *in vivo*. Subsequent transplantation into the SCI site of 6–8 month old canines resulted in significant motor recovery of paralyzed pelvic limbs, where there storation of neural relays restored information to the paralyzed hind limbs (Lai et al., 2019). New stem cell neurotechnologies can be adopted to refine this approach by instead producing the precise therapeutic cells *in vitro*, similar to neural ribbons (Olmsted et al., 2020, under review). Syngeneic and allogeneic iPSC-derived NSPCs were shown to survive after transplantation into the spinal cord of minipigs (Strnadel et al., 2018). In a first-in-human Phase 1 SCI clinical trial for chronic SCI, clinical grade NSCs from line NSI-566 were transplanted as a part of a safety analysis for treatment of clinically complete thoracic transections (Curtis et al., 2018). An alternate Phase 1 trial using autologous adipose tissue-derived MSCs for traumatic SCI was also reported by the Mayo Clinic (Bydon et al., 2019). In these two recent works, the stem cell therapies were deemed safe, but meaningful clinical improvement in function was not observed. Interestingly, by incorporating collagen scaffolds adsorbed with patient autologous MSCs into the transplantation site, two traumatic SCI patients regained motor, sensory, and autonomic functions one year later in a separate study (Xiao et al., 2018). Currently, a variety of stem cell based sources of cells and protocols are being used, with varying degrees of success. The field may benefit by unifying efforts to focus on those most promising neural or MSC therapies. It is also evident that a combination of biomaterials with stem cell-derived neural cells adds further benefit to rehabilitation regimens (Harkema et al., 2011) along with spinal cord stimulation to drive neuroplasticity after transplantation (Formento et al., 2018). Human SCI clinical trials with cell-based therapies were recently reviewed (Silvestro et al., 2020; Yamazaki et al., 2020), along with a protocol in place for systematic review and meta-analysis of efficacy (Tang et al., 2020). An excellent report on the requirements for translation of stem cell therapies to the clinic for CNS repair including industry and regulatory considerations is provided by Aboody et al. (2011).

## Conclusions and Future Perspectives

**Figure 5:**
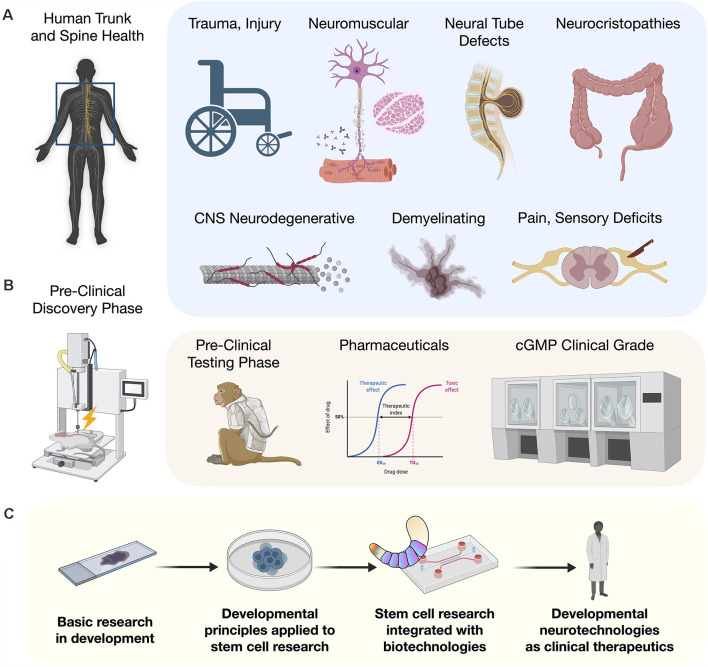
Clinical potential of stem cells and developmental neurotechnologies. **(A)** Conditions of the human trunk and spine that will benefit from developmental neurotechnologies. Examples include trauma and spinal cord injury (SCI), neuromuscular disorders such as amyotrophic lateral sclerosis (ALS) and myasthenia gravis, neural tube closure defects such as spina bifida that relate to PAX3, neurocristopathies such as Hirschsprung’s disease that result from NCC migration defects, CNS neurodegeneration, demyelinating disorders such as multiple sclerosis, and sensory disorders such as neuropathic pain. **(B)** Clinical stages of developmental neurotechnology discovery and testing from pre-clinical through to current good manufacturing processes (cGMP). **(C)** The developmental neurotechnology paradigm to generate clinical therapies and disease models by merging stem cell developmental principles with neurotechnologies.

The first decade of the 21st century has seen a new revolution in human developmental neurotechnologies that would not be possible without mouse and now human stem cell advances that continue to accelerate (Figure 5; Thomson et al., 1998; Takahashi and Yamanaka, 2006; Cong et al., 2013; Mayr et al., 2019). It is now primarily the temporal nature of clinical trials, cGMP, and natural healing that will determine the timeframe to therapeutic advancements. For Parkinson’s disease, clinical trials by GForce-PD cell replacement therapies have recently begun (Barker et al., 2017). Not only the stem cell based models are bringing expanded understanding of fundamental elements of human disease processes, but also the temporal scope around disease initiation and progression, while also doubling as treatments testing “bench-to-therapy” platforms. Stem cell neurotechnologies for injury and trauma have advanced in part through more complex integration with biomaterials, optogenetics, biosensors, and electronics and are pushing the boundaries in research discovery and treatment. Specifically for human therapies of the trunk and spine (Figures 4, 5), information arising from developmental neurotechnologies, including embryology, can now be analyzed to inform and improve treatments beyond trauma. Most recently, this includes neuromuscular disorders such as ALS and myasthenia gravis (reviewed in Sances et al., 2016; Faustino Martins et al., 2020), neural tube defects such as spina bifida that can be related to the *Pax3* gene (Sudiwala et al., 2019), neurocristopathies such as Hirschsprung’s disease resulting from NCC migration defects (Fattahi et al., 2016; Workman et al., 2017), neurodegeneration or demyelinating process originating in the CNS (Garden and La Spada, 2012), and sensory disorders such as neuropathic pain (Manion et al., 2020). We expect that human stem cell developmental neurotechnologies will continue to play an essential role in understanding and treating these disorders through the elementary discovery of fundamental processes, *in vitro* models, and scalable high throughput platforms for drug testing, as well as by supplying an unlimited customized cell resource for replacement therapies. A summary of select recent stem cell models applied to disease and therapeutic studies is provided (Table 1). Developmental neurotechnologies are further benefiting by the use of population diverse hiPSC lines (Chang et al., 2015; Tomov et al., 2016; Olmsted and Paluh, 2020) to more accurately represent global ethnicities and genomes (Laurent et al., 2010; Mosher et al., 2010; Gurdasani et al., 2019).

**Table 1:** Stem cell technologies for trunk and spine disease modeling and therapy (select publications).

Studies	Cell source	Lineage(s)	Disorder	Category	Model
Lee et al. (2012)	hiPSC	NCC to autonomic neurons	Familial dysautonomia	Neurocristopathy	Therapeutic screen
Mica et al. (2013)	hESC, hiPSC	NCC to melanocytes	Pigmentation defects	Neurocristopathy	Stem cell
Fattahi et al. (2016)	hESC, hiPSC	NCC to enteric neurons	Hirschsprung’s disease	Neurocristopathy/ congenital	Cell therapy, drug discovery
Workman et al. (2017)	hESC, hiPSC	NCC to enteric neurons	Hirschsprung’s disease	Neurocristopathy/ congenital	Organoid, *in vivo*
Chen et al. (2014), Kiskinis et al. (2014), Wainger et al. (2014) and Sances et al. (2016) review	hiPSC	Motor neurons	SOD1 ALS	Neuromuscular/ neurodegenerative	Stem cell
Sareen et al. (2013) and Devlin et al. (2015)	hiPSC	Motor neurons	C9ORF72 ALS	Neuromuscular/ neurodegenerative	Stem cell
Osaki et al. (2018)	hESC, hiPSC	Motor neurons	Sporadic ALS	Neuromuscular/ neurodegenerative	Device
Bryson et al. (2014)	mESC	Motor neurons	Denervation	Neuromuscular/ neurodegenerative	Therapy
Hor et al. (2018) and Adami and Bottai (2019) review	hiPSC	Motor neurons	SMA	Neuromuscular/ neurodegenerative	Organoid for therapeutic testing
Steinbeck et al. (2016)	hiPSC	Motor neurons	Myasthenia gravis	Neuromuscular	Co-culture
Faustino Martins et al. (2020)	hESC, hiPSC	Spinal neurons, skeletal muscle	Myasthenia gravis	Neuromuscular	Organoid
Chal et al. (2015)	mESC	Skeletal muscle fibers	Duchenne muscular dystrophy	Neuromuscular	Stem cell
Maffioletti et al. (2018)	hESC, hiPSC	Skeletal muscle	Multiplemuscular dystrophies	Neuromuscular	Tissue engineering
Mazzara et al. (2020)	hiPSC	Peripheral sensory neurons and glia	Friedreich’s ataxia	Sensorimotor	Organoid
Dionisi et al. (2020)	hiPSC	Primary proprioceptive neurons	Friedreich’s ataxia	Sensorimotor	Stem cell
Xiong et al. (2021)	hPSC	DA neurons	Parkinson’s disease	Neurodegenerative	Therapy
Tao et al. (2021)	Autologous monkey	DA neurons	Parkinson’s disease	Neurodegenerative	Therapy
Fandel et al. (2016) and Manion et al. (2020)	hESC, hiPSC	Interneuron precursors, GABAergic interneurons	Neuropathic pain	Pain/Sensory	Therapy
van den Brink et al. (2020)	mESC	Neural tube, somites	Embryonic mutagenesis	Congenital	Gastruloid
Veenvliet et al. (2020)	mESC	Neural tube, somites	Embryonic mutagenesis	Congenital	Gastruloid
Lee et al. (2020)	Human fibroblasts	Induced motor neurons	SCI	Traumatic	Therapy
Xiao et al. (2018)	MSC	MSC	SCI	Traumatic	Therapy

Towards the goal of reproducible human SCI transplantation therapy, the field is embracing regional matching of therapeutic cells.This is now possible by the wealth of studies with stem cell-derived neural cells, such as described in this review, that reveal a comprehensive understanding of key signaling pathways and complex, intrinsic gene regulatory networks. Organoid, gastruloid, assembloid, and circuitoid technologies reveal the inherent ability of cells to adapt into functional complexes. Matched therapeutic cells by definition should interface more appropriately and efficiently with host. Biomaterial platforms that facilitate better cell–cell interactions and favor network connectivity *in vitro* are an elegant approach to bridge *in vitro* design with *in vivo* transplantation (Lai et al., 2019) and are continuing to evolve (Olmsted et al., under review). Neuronal maturation, synaptogenesis, and plasticity are also driven by network synchronous-firing activity (Kirkby et al., 2013) i.e., optimal in 3D vs. adherent neuronal cultures. Neuromodulation by use of programmed, electrical frequency-specific stimulation of the dorsal columns has been demonstrated to be critical for managing chronic pain patients that are refractory to medical management (Staudt et al., 2020), and may be needed in developing more complex *in vitro* systems to drive appropriate network formation and synaptic plasticity mechanisms for motor recovery. Continually evolving and integrating methodologies and tools to elucidate, and exploit natural mechanisms required for functional maturation will continue to position the field within an exciting era of innovative reproducible treatments.

In closing, stem cell developmental neurotechnologies will continue to be cross-disciplinary, bringing together additional fields beyond stem cell biology, neurodevelopment, multi-omics, and bioengineering. To this end, recent advancements in genetic engineering of human cell lines enabled by CRISPR/Cas gene-editing technologies are advancing differentiation, enrichment, subtype purification, comparison of cell types in normal and disease states, and assessment of functional graft integration (Cota-Coronado et al., 2019; Tian et al., 2019; Paschon et al., 2020). Additionally, 3D organoid and complex co-culture platforms have improved context-specific maturation of multiple neural cell types. The use of pharmacological agents, targeting the cytoskeleton and consideration of innate circadian clocks have also contributed to an understanding of *in vitro* maturation in the stem cell field (Alvarez-Dominguez et al., 2020; Hogrebe et al., 2020) and are now extending to protocols using NMPs. Furthermore, sophisticated co-cultures of PSC-derived neurons with astrocytes have produced marked improvement in population-level connectivity and synaptogenesis in addition to more hyperpolarized resting membrane potentials (Taga et al., 2019). The ability to generate functionally mature neurons *in vitro* along with other cell types that are indistinguishable from *in vivo* counterparts is unlikely to be required for all therapeutic success, particularly for simpler injuries and microenvironments that allow cells to mature quickly after transplantation. However, it is likely necessary *in vitro* to accurately model disease mechanisms, and will benefit pharmaceutical development to ultimately guide appropriate *in vivo* transplantation and non-transplantation therapeutic interventions alike.

## Author Contributions

ZO composed figures. All authors contributed to the article and approved the submitted version.

## Conflict of Interest

The authors declare that the research was conducted in the absence of any commercial or financial relationships that could be construed as a potential conflict of interest.
